# For long-term sustainable software in bioinformatics

**DOI:** 10.1371/journal.pcbi.1011920

**Published:** 2024-03-15

**Authors:** Luis Pedro Coelho

**Affiliations:** 1 Centre for Microbiome Research, School of Biomedical Sciences, Queensland University of Technology, Translational Research Institute, Woolloongabba, Queensland, Australia; 2 Centre for Data Science, Queensland University of Technology, Brisbane, Australia; University of Virginia, UNITED STATES

This is a *PLOS Computational Biology* Benchmarking paper.

## Introduction

Research software has become essential in science, including in the life sciences [1,2]. Most of this software is designed and built by research groups funded by short-term research grants. The coding is performed by trainees who move on to other jobs once the project is completed. This can result in dead software tools, whereby a tool described in a manuscript no longer works after publication [3,4]. Commercial software uses sales to pay for long-term maintenance and support. In addition to the financial costs, commercial software often lags behind the capabilities of academic software—a natural consequence of the fact that new ideas are often explored first in academia—and commercial software may not be open source, which poses a barrier for some researchers as it can hinder reproducibility. It is noteworthy, though, that, in the field of machine learning, it is not uncommon for commercial entities to release high-quality tools, including AlphaFold2 [5,6], as well as libraries such as PyTorch [7], which then are incorporated by academic research labs into their computational biology projects.

During my training and now, as a group leader, I have created several open source research software tools. My group publicly commits to maintaining these for a minimum of 5 years after the publication of the associated manuscript. In practice, several of our projects have been maintained for longer given that they continue to be used. Ideally, software should be maintained for as long as it is being actively used. We also provide some basic support to users, in the form of addressing their queries. In this perspective, I will first define exactly what is meant by *maintenance* and when we are committing to it, then describe how we make it possible, and finally end with a call for the community and institutions to be explicit about what users can expect from each piece of research software.

*Maintaining* software is different from *extending it*. Although the exact boundary between the 2 activities is not always clear cut; maintenance, to me, means that the software should perform as described in the manuscript. This means fixing any bugs that are reported. This may mean that changes are necessary, for example, to accommodate new versions of dependencies which break backwards compatibility (a common cause of software collapse [3]). If the software is provided as a service (e.g., as a web server), concerns related to security and privacy may lead to required updates and even lower-level network functionality may need to be updated to keep the service operational. All of the practices I will describe make it easier to extend the code to perform beyond what is described in the manuscript, but we do not commit to this.

I also wish to distinguish between the code that we make available as *Tools* for others and code that we make available to support papers whose main purpose is to present biological findings. Both types of code play an important role in research and both should be made available. Given their different purposes, however, they are subject to different tradeoffs (see Box 1) and researchers should make it clear what is the purpose of the code they publish.

**Box 1: Different levels of research software**

**Level 0: One-off analysis**. This comprises the day-to-day development of scripts. These will be limited in scope to the current application and it is accepted that they will often contain minor bugs such as mishandling boundary conditions. Part of the process of computational science is to find (and correct) these errors to prevent erroneous conclusions. Most analyses will only be intermediate steps towards a publication and will be discarded *en route*.

**Level 1: *Extended Methods Code* (for support of a results-driven publication)**. When we publish a paper that focuses on biological results, we will publish the analysis scripts alongside it. The goal is to act as an extension to the Methods Section, disambiguating any unclear statements made in natural language, while also making it easier for others to reproduce and build on the work. However, we expect that only a small minority of the readers of the paper will use (or even download) the code. While this code generally started as *one-off analysis code* (Level 0), it was reviewed for potential flaws. Furthermore, we added a modicum of documentation and took care to ensure that the code is understandable by an outside reader and that it does not rely on the specific installation and environments we use (e.g., no internal paths). Nonetheless, this code produces a single result and is not primarily intended to be used by others.

**Level 2: A *Tool* for others to use**. A tool for others to use is the most demanding type of code that we produce. Here, the goal is that the highest possible number of readers of the paper become users of the tool. For long-term robustness, it is important that such Level 2 code be as user-friendly, well-documented, and platform-independent as possible.

Code that was originally intended for a single project (Level 1) can be upgraded to a *Tool*, but it requires effort. For example, *Extended Methods Code* is often tested in an ad hoc fashion, but we always implement automated tests for *Tools*. As a rule of thumb, taking code from one level to the next requires a 5- to 10-fold increase in coding effort. I estimate this higher than the 3-fold rule that Fred Brooks proposed in an analogous setting in software development [18] as we have made significant strides in efficiency when writing analysis code, without concomitant developments in generating tests and documentation.

It is just as ill-advised to release under-engineered software as a *Tool* (thus, misleading potential users as to the quality of the underlying code) as to expend resources to optimize and make robust code that will only be used a small number of times. As described in the main text, we aim to have tools that produce high-quality error messages by detecting cases such as insufficient temporary disk space. It could be wasteful, however, to expend the effort necessary to add this functionality to *Extended Methods Code*.

In this context, there is extensive literature on reproducibility of computational results [8,9] and recommendations overlapping with our practices [10,11]. Most often, the focus has been on what I term *Extended Methods Code* (Level 1; see **Box 1**). However, this is a different challenge from the one that I address here, as I focus on code that implements *Tools* (Level 2; see **Box 1**). In fact, I consider that conflating these 2 classes of code deliverables (both of which are common in bioinformatics and computational biology) has hampered progress towards higher standards. Internally, in my group, I have found it very helpful to explicitly establish when there is an expectation that effort will be put into the code to make it widely applicable (a *Tool*) and when it is tolerable that the code be less robust (although, naturally, still correct in its limited domain). Nonetheless, there are linkages between the reproducibility of analysis code and tool maintenance: on the one hand, reproducible research methods are the baseline for our work; on the other hand, tools no longer working is one of the reasons that results stop being reproducible [12,13] so that long-term tool maintenance will contribute to diminishing the reproducibility problem.

These distinctions were developed internally in my group, but they mirror the first 3 levels of Hong and colleagues [14]. The more complex levels they consider are those that go beyond the small projects considered here and include critical tools such as BLAST [15] or STRING [16]. For those projects, with millions of users and thousands of yearly citations, the strategies I present here will not scale. However, smaller projects, developed by a single lab, constitute the majority of bioinformatics work and are much less likely to be able to attract dedicated funding for dedicated Research Software Engineers [17].

## How we develop software that is maintainable over the long-term

Having established that our commitment is to maintaining and supporting code that we release as *Tools*, I will now describe 7 practices we use to achieve it. Although the commitment is forward-looking, most of its effect is to change our behavior *prior to* and *immediately after* publication. The goal is that the software continues to be available and useful while minimizing the long-term effort from the part of the authors and minimize the disruption when the main author of the tool leaves the group.

### We employ the methods of reproducible research

Techniques for reproducible research are most often presented as enabling other research groups to reproduce specific analytical results, focused on what I have called *Extended Methods Code*. We have found that they are also useful for reproducible research *within a single lab*. Furthermore, many of the same approaches are applicable to code developed as a *Tool*. Practices such as using automated version control are essential for developing any research code [19] and assist with long-term maintenance. Automated testing in particular [20] is incredibly useful at ensuring that the code continues to work when new versions of dependencies are available as one can quickly check whether something is broken by running the test suite on the new version.

### We eat our own dogfood

Since my group works on both tool development and projects that are driven by biological questions, we use our tools as well, a practice known as dogfooding (from the expression “to eat your own dog food”). Testing software while performing tasks that are not specifically focused on testing, called *implicit testing* [21], finds many issues, particularly when there is an expectation that issues found will be reported and fixed (rather than simply worked around).

One of the difficulties in providing tools is that one loses the perspective of how a beginner approaches the problem. Therefore, we ask that incoming students attempt to install any software using only publicly available sources and report any issues. It is important to communicate to students that finding faults with documentation is an important outcome of their efforts in order to avoid that they take shortcuts that are not available to the wider community (most obviously, asking the original authors of the tools for offline help).

### We follow the principle of least astonishment in design

This is a common user interaction principle, whereby one attempts to minimize unexpected behaviors. When it comes to tools, it often means using standard interfaces like those found in other tools (e.g., using the same option names in the case of a command line tool) [22]. Deviations from standards—even when documented—not only lead to a worse user experience, but also can cause excessive support requests from the confused users. Even the choice of GitHub is informed by the fact that it is the most popular code sharing platform, including in bioinformatics [23], and thus users are most likely to already have active accounts on the platform and be familiar with it, which encourages engagement.

### We provide support in public

As users often face similar issues, it is inefficient to tackle each of them individually, in a one-off manner. In particular, providing support by private email benefits only the immediate recipients, even as others (perhaps in the future) are likely to encounter the exact same issue. Therefore, we try to provide support using public fora, such as a mailing-list, GitHub Issues, or YouTube videos. When answering a single individual, we often also copy the response into the documentation.

To achieve this, you need to first make it clear to users what the preferred support channels are. When users contact us directly by email, we steer them towards the public forum. This strategy is also employed by corporations that provide services free of charge and provide support only on forums where a significant fraction (often the majority) of the support is provided by other users.

### We aim to have our software produce high-quality error messages

A high-quality error message is one that enables users to quickly diagnose and, if possible, fix the underlying issue. Where possible, we treat confusing error messages as bugs. Good error messages not only provide a better user experience, but they can reduce the amount of support that needs to be provided.

For example, when a user reports a long and confusing error that results from a lack of disk space, it is tempting to simply reply that they need to increase their temporary storage capacity. However, when feasible, we also add the extra code to detect and report this situation. A good message would read like “not enough temporary disk space in temporary directory: /tmp”—note that by explicitly printing the temporary directory being used, we also help the user identify the case where the temporary directory has been misconfigured. We hope that this will enable the next user facing the same problem to solve it by themselves without the need to contact us.

It is possible to systematically test for these issues by running tests in artificially constrained environments, but, in practice, we rely on user reports.

### We provide bioconda packages [24] for all our tools

Providing bioconda packages almost completely eliminates bug reports related to installation, which were very frequent prior to the widespread use of bioconda. It also makes it easier to reliably reuse other tools. For *Extended Methods* (Level 1) code, the reuse of external code is almost always a positive, but when it comes to *Tools* (Level 2), reusing code also introduces a dependency, which imposes costs [11]. By making bioconda installation the preferred method, we can ensure that users have a simple way to install all necessary dependencies.

### We make our software available prior to publication

Beta users are incredibly important for catching bugs, including usability and documentation bugs. Thus, we make our software available even prior to publication and advertise it online and at conferences. We often attract testing users who report bugs and other issues.

## Discussion and conclusions

I presented several principles that I use in my group to provide long-term support for the tools we publish at minimal long-term cost. A common theme is to not only fix any immediate problem encountered or reported, but also to attempt to avoid similar issues in the future or to equip users to solve them themselves. Often, when using a platform such as Github Issues, the user reporting the original bug will close the issue as soon as their problem is solved. While this is well-intentioned, I will reopen the issue with a note that there is a possible improvement to the software (for example, that an error message could have provided more information) and only close the issue once this improvement has been implemented.

When we first release a public test version, and immediately after the manuscript is published, users will report many issues, including lacuna (and outright mistakes) in the documentation. There is a risk that junior students get discouraged if they perceive this user feedback as negative critiques of their work. It is important to stress that receiving bug reports is actually a very positive sign as it indicates engagement and leads to improvement of the code. As reported issues get tackled, the stream of reports should slow down even as usage continues to increase. Questions are also easier to handle when we can point users to previously written documentation rather than having to write an individualized answer.

The perception that bioinformatics software is low-quality damages the reputation of the field as a whole. Therefore, I hope that others will copy the commitment that my group makes. I further hope that similar pledges become institutionalized: funders (including peer reviewers evaluating grant proposals) should evaluate the track record of applicants in keeping their software up-to-date while considering whether to provide new funding.

For journals, my suggestion is that journals should demand a Maintenance and Support statement. This is modeled on the Data Availability statement that is already required by many venues as part of data sharing mandates. Although compliance with data sharing mandates is not perfect, sharing has improved over time [25–29] and I hope for similar results for software Maintenance and Support. If authors of a new algorithm use the Maintenance and Support Statement to declare that they are making code available only as a proof-of-concept without any commitment to support, this would make it clear to readers that some effort may be required to use the code and give space for others to reimplement the approach in a user-friendly tool. If authors declare that they are maintaining the code for a certain number of years (I suggest 5 as a reasonable default expectation), then users would be empowered to ask for support and updates.
